# CD37 high expression as a potential biomarker and association with poor outcome in acute myeloid leukemia

**DOI:** 10.1042/BSR20200008

**Published:** 2020-05-27

**Authors:** Qi Zhang, Qi Han, Jie Zi, Chunhua Song, Zheng Ge

**Affiliations:** 1 Department of Hematology, Zhongda Hospital, Medical School of Southeast University, Institute of Hematology Southeast University, Nanjing 210009, China; 2 Department of Pediatrics, Pennsylvania State University College of Medicine, Hershey, PA 17033, U.S.A.

**Keywords:** acute myeloid leukaemia, CD37, gene expression, prognosis

## Abstract

**Background:** CD37, a member of the transmembrane 4 superfamilies (TM4SF), has been proved to be abnormally expressed in a range of malignancies. Herein, we investigate the effects of CD37 expression and analyze its clinical outcome in acute myeloid leukemia (AML) patients.

**Methods:** The RNA-seq and clinical data of AML patients were obtained from cBioPortal database. CD37 correlated genes, the expression prolife and survival curve of eight key genes were acquired from Gene Expression Profiling Interactive Analysis (GEPIA) and UALCAN. Pathway enrichment and protein–protein interaction (PPI) network analysis were performed based on metascape databases.

**Results:** Our results showed that CD37 mRNA expression level was significantly up-regulated in patients with AML compared with healthy persons. Patients with high CD37 expression had shorter overall survival (OS) and disease-free survival (DFS). Pathway analysis data showed that CD37 is involved in DNA replication, RNA transport, Salmonella infection, ribonucleoprotein complex biogenesis, cell cycle phase transition and so on. Furthermore, we found eight genes correlated with CD37 are all highly expressed in AML patients, and high expression is associated with poor prognosis.

**Conclusion:** Our study described systematical expression profiles and the prognostic values of CD37 in AML; our data suggested CD37 might be novel therapeutic target and promising prognostic biomarker in the patients.

## Background

It started a new era when the first monoclonal antibody (mAb), known as rituximab, for treatment of low-grade non-Hodgkins lymphoma was approved by the Food and Drug Administration (FDA) in 1997 [1]. Since then, many other monoclonal antibodies like anti-CD19, anti-CD22, anti-CD23 and anti-CD80 have also been studied. In the late 1980s and early 1990s, the anti-CD37 antibody was used for radioimmunotherapy [2,3].

CD37 belonged to TM4SF (transmembrane 4 superfamily), which was characterized by four potential membrane-spanning regions [4,5]. The CD37 antigen was strongly expressed on B lymphocytes with positive surface immunoglobulin, but it was weakly expressed on a subpopulation of monocytes, granulocytes and T cells, whereas neither platelets nor red blood cells expressed CD37 [6,7]. In terms of cancers, deficiency of CD37 induced the development of B-cell lymphoma [8]. In addition, anti-CD37 agents were clinically active and well tolerated in patients with chronic lymphocytic leukemia (CLL) according to preclinical and early clinical studies [9]. However, the research about CD37 in acute myeloid leukemia (AML) was very limited.

AML is a highly heterogeneous group of diseases characterized by infiltration of the myeloid blasts in bone marrow, peripheral blood or other tissues [10]. It is the most common form of acute leukemia among adults. In 1990, there were 0.96 deaths per 100000 because of AML worldwide, and by 2017 it has increased to 1.31 [11]. The general therapeutic strategy of AML includes induction chemotherapy and consolidation therapy. But if patients do not get poster-mission therapy, they will experience relapse within 6–9 months [12]. Besides, toxicity and morbidity caused by chemotherapy are fatal, especially for elderly patients. Therefore, it is of great urgency to identify reliable biomarkers, enable early diagnosis, improve prognosis, develop new therapeutic strategies.

## Methods

### TCGA data and the cBioPortal

The cBioPortal is an open-access resource that provides a visual tool for researching and analyzing cancer gene data. Up to now, it offers data on more than 5000 tumor samples from 20 studies [13]. In this study, we obtained CD37 mRNA expression z-scores (RNA Seq V2 RSEM) and detailed information of patients with AML.

### The Gene Expression Profiling Interactive Analysis dataset

Gene Expression Profiling Interactive Analysis (GEPIA) is a tool which delivers integrated information to end users, including differential expression analysis, profiling plotting, correlation analysis, patient survival analysis, similar gene detection and dimensionality reduction analysis [14]. We performed CD37 mRNA expression levels in various cancers, the differential expression of CD37 genes in AML and healthy donor samples, survival analysis between high CD37 TPM (transcripts per million) and low CD37 TPM.

### UALCAN

UALCAN is an online platform that makes an in-depth analysis of TCGA gene expression data [15]. It is able to assess the effect of gene expression levels and clinicopathological features on patient survival. In the current study, UALCAN was applied to analyze the association between the transcriptional levels of CD37 and clinical outcome. Genes co-expressed with CD37 were also analyzed via UALCAN.

### The Cancer Cell Line Encyclopedia dataset

The Cancer Cell Line Encyclopedia (CCLE) assembles 947 human cancer cell lines, covered gene expression, chromosomal copy number, abundant parallel sequencing data and pharmacologic profiles of 24 anticancer drugs across 479 cancer cell lines [16]. By using it, we gained the CD37 expression levels in 14 AML cell lines.

### Metascape

Metascape is a powerful and efficient tool, the purpose of which is designed to supply a comprehensive gene list annotation as well as analysis resource for users [17]. Metascape can help users to apply the current popular bioinformatics analysis methods to realize the understanding of genes or protein functions.

### DiseaseMeth

DiseaseMeth collects experimental information from over 14000 entries and 175 high-throughput datasets from a wide number of sources. It provides integrated gene methylation data based on cross-dataset analysis for disease and normal samples [18].

## Results

### CD37 expression in patients with AML

**Figure 1:**
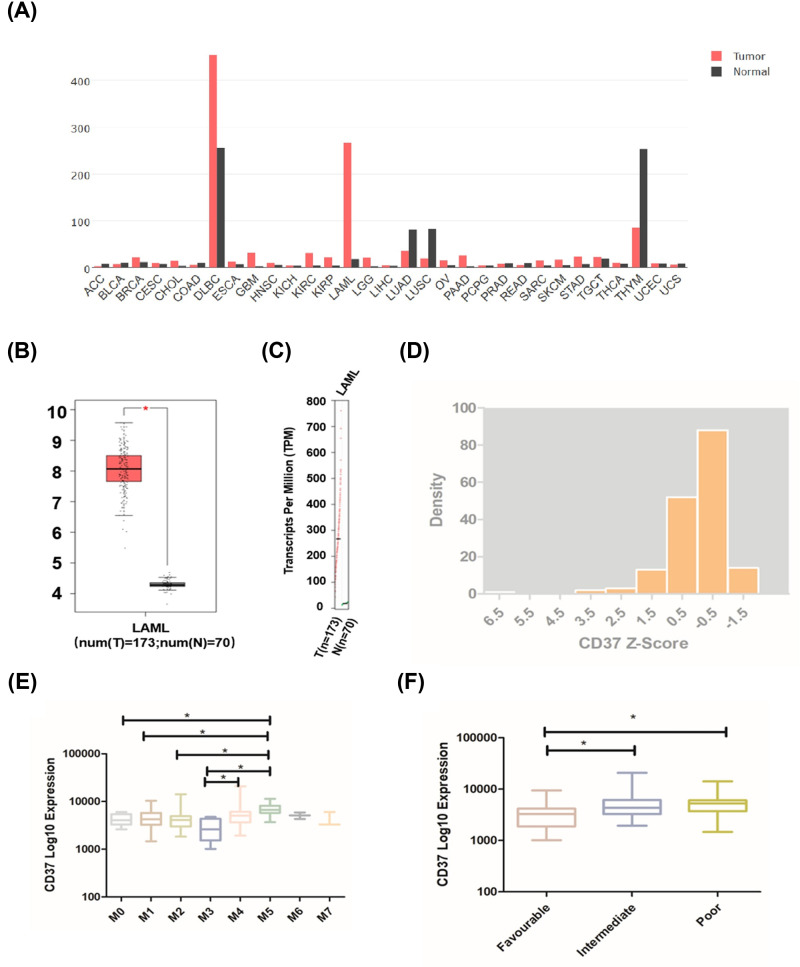
CD37 expression levels in different types of cancers (**A**) The expression of CD37 in pan-cancer by GEPIA. (**B**) CD37 expression in AML compared with healthy control. (**C**) The expression level of CD37 in AML. (**D**)The distribution of CD37 Z-score. The distribution of CD37 log10 mRNA expression classified by (**E**) FAB norm; (**F**) risk status. *P<0.05.

The mRNA levels of CD37 in different cancers were obtained from GEPIA database. As shown in Figure 1A, CD37 has the highest mRNA level in diffuse large B-cell lymphoma (DLBCL) and ranks second in AML. Furthermore, the results showed that CD37 is highly expressed in patients with AML compared with a healthy person (Figure 1B,C). These data suggested that CD37 plays a potential role in AML. We next downloaded CD37 expression data from cBioPortal. When CD37 Z score is between 0 and −1, the number of patients is the largest, and between 6 and 7 is the least (Figure 1D). To figure out whether there was significant difference in CD37 expression among patients classified by The French–American–British (FAB) classification systems, we summarized the expression level of each subgroup (Figure 1E). CD37 expression is relatively higher in M5, which is significantly different from that in M0, M1, M2 and M3 (M0: *P*=0.017; M1: *P*=0.008; M2: *P*=0.007; M3: *P*=0.000013). We also observed that CD37 expression is relatively lower in M3 but it is only significantly different from M5 and M3 (M3 vs M4: *P*=0.016). We analyzed CD37 expression in different risk groups based on NCCN AML classification (Figure 1F), and found that the CD37 expression is significantly lower in favorable risk group than that of intermediate and poor risk group (intermediate: *P*=0.002; poor: *P*=0.000344). The patients from TCGA datasets were further divided into CD37 high (Z score ≥ 1, *n*=19) and low (Z score < 1, *n*=154) group. No significant difference was observed in the two groups on patients’ age, white blood cell counts, percentages of bone marrow blasts, etc. (Table 1). Also, no significant association was identified between the level of CD37 expression and the mutations of IDH1, IDH2, RUNX1, TET2, NRAS, CEBPA, WT1, DNMT3A, NPM1 and TP53 (Table 2).

**Table 1:** The clinical characteristics of patients with AML from cBioPortal

Parameter	Total	CD37 low (Z < 1)	CD37 high (Z > 1)	*P*-value
**Male (%)**	92 (53.1)	84 (91.3)	8 (8.70)	0.338
**Median age (y)**	58	57	63	0.147
**Median WBC**	17	14.9	34.8	0.116
**Median PB**	39	41	22	0.171
**Median BM**	72	73	63	0.129
**NCCN subtype**				vs favorable
Favorable	33	32	1	
Intermediate	92	82	11	0.182
Poor	45	37	7	0.128
**FAB subtype, number**				
M0	16	16	0	
M1	44	40	4	
M2	38	35	3	
M3	16	16	0	
M4	34	29	5	
M5	18	11	7	
M6	2	2	0	
M7	3	3	0	

**Table 2:** The relation of CD37 expression and common mutation in AML

	Total	CD37 low (Z < 1)	CD37 high (Z > 1)	*P*-value	
				CD37 low vs. high	Fisher exact test
**FLT3, number (%)**				0.837	1
Present	49 (28.32)	44 (89.80)	5 (10.20)		
Absent	124 (71.67)	110 (88.71)	14 (11.29)		
**IDH1, number (%)**				0.526	1
Mutated	16 (9.25)	15 (93.75)	1 (6.25)		
Wild-type	157 (90.75)	139 (88.54)	18 (11.46)		
**IDH2, number (%)**				0.914	1
Mutated	17 (9.83)	15 (88.24)	2 (11.76)		
Wild-type	156 (90.17)	139 (89.10)	17 (10.90)		
**RUNX1, number (%)**				0.914	1
Mutated	17 (9.83)	15 (88.24)	2 (11.76)		
Wild-type	156 (90.17)	139 (89.10)	17 (10.90)		
**TET2, number (%)**				0.577	1
Mutated	15 (8.67)	14 (93.33)	1 (6.67)		
Wild type	158 (91.33)	140 (88.61)	18 (11.39)		
**NRAS, number (%)**				0.075	0.13
Mutated	12 (6.94)	9 (75)	3 (25)		
Wild-type	161 (93.06)	145 (90.06)	16 (9.94)		
**CEBPA, number (%)**				0.599	0.638
Mutated	13 (7.51)	11 (84.62)	2 (15.38)		
Wild-type	160 (92.49)	143 (89.38)	17 (10.63)		
**WT1, number (%)**				0.254	0.604
Mutated	10 (5.78)	10 (100)	0 (0)		
Wild-type	163 (94.22)	144 (88.34)	19 (11.66)		
**DNMT3A, number (%)**				0.603	0.784
Mutated	45 (26.01)	41 (91.11)	4 (8.89)		
Wild-type	128 (73.99)	113 (88.28)	15 (11.72)		
**NPM1, number (%)**				0.349	0.416
Mutated	48 (27.75)	41 (85.42)	7 (14.58)		
Wild-type	125 (72.25)	113 (90.4)	12 (9.6)		
**TP53, number (%)**				0.633	1
Mutated	14 (8.09)	13 (92.86)	1 (7.14)		
Wild-type	159 (91.91)	141 (88.68)	18 (11.32)		

### CD37 expression in AML cell lines

**Figure 2:**
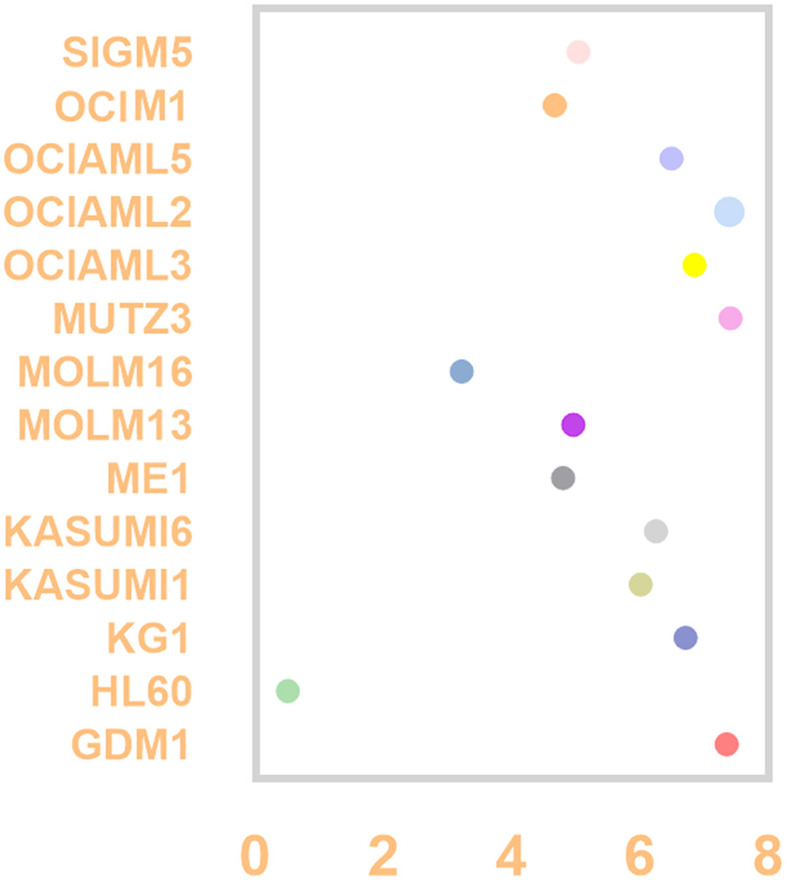
CD37 expression level in AML cell lines by CCLE

Using the CCLE dataset, we compared the CD37 mRNA level identified by RNA-seq in AML cell lines which include GDM-1, HL-60, KG-1, Kasumi-1, Kasumi-6, ME-1, MOLM-13, MOLM-16, MUTZ-3, OCI-AML3, OCI-AML2, OCI-AML5, OCI-M1 and SIG-M5. The top three cell AML lines with the highest expression are MUTZ-3, OCI-AML2, GDM-1, and the lowest is HL-60 (Figure 2).

### Prognostic values of CD37 in AML

**Figure 3:**
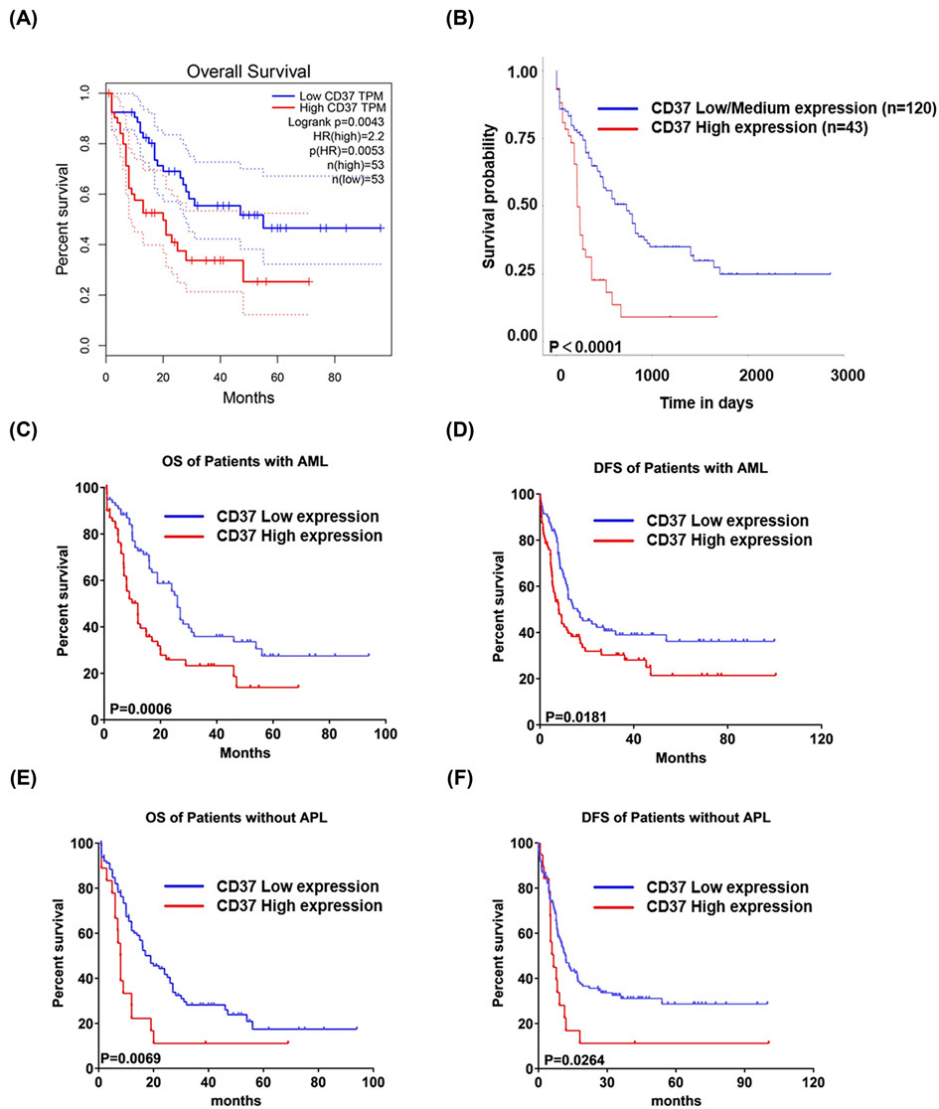
The survival analysis of CD37 in patients with AML (**A,B**) The survival curves of OS in AML patients with regard to CD37 expression by GEPIA and UALCAN, respectively. (**C,D**) The OS and DFS of patients with AML categorized by CD37 median expression. (**E,F**) According to CD37 Z-score, the OS and DFS of patients without APL.

The prognostic role of CD37 in AML was further explored by means of GEPIA and UALCAN databases, and the significant association of CD37 high expression with poor overall survival (OS) in AML was observed in both databases (Figure 3A,B). To further validate the association between CD37 expression level and clinical outcome, we also dichotomized patients into high and low CD37 expression groups by CD37 median expression. A similar trend was observed that patients with high CD37 expression had significantly shorter OS as well as disease-free survival (DFS) than CD37 low expression patients (OS-median: 11.99 vs. 26.02 months, *P*=0.0006; DFS-median: 8 vs.16.1 months, *P*=0.0181; Figure 3C,D). As is known to all, acute promyelocytic leukemia (APL) is a particular subtype in AML, characterized by a balanced reciprocal translocation between chromosomes 15 and 17, which leads to the fusion between promyelocytic leukemia (PML) gene and retinoic acid receptor α (RARα) [19]. The good news is the complete remission (CR) rate was increased to 90–95% and 5-year DFS to 74% because of all-*trans-*retinoic acid (ATRA) or the ATRA-based regimens [20]. For these reasons, we excluded the patients of M3 from the survival analysis. Yet, the OS and DFS of CD37 high group (Z ≥ 1) is still shorter than the low group (OS-median: 7.965 vs. 18.96 months, *P*=0.0069; DFS-median: 6.6 vs. 11.9 months, *P*=0.0264; Figure 3E,F). Univariate analysis showed that high CD37 mRNA levels were associated with increasing age, poor molecular risk, transplant status, white blood cell count. This association was confirmed in multivariate analyses except for transplant status (Table 3).

**Table 3:** Multivariate Cox regression model between OS in AML and other clinical parameters

Characteristic	*CD37* high expression (*n*=19)	*CD37* low expression (*n*=154)	Univariate analyses (Chi-square tests)	Multivariate analyses (Multivariate Cox model)	
			*P*-value	*P*-value	HR (95% CI)
Age (y; median; range)	63 (31–88)	57 (18-82)	<0.001	<0.001	1.041 (1.023–1.058)
Male (%)	42.1	53.2	0.858	0.793	0.948 (0.637–1.412)
WBC, ×10^9^/l (median; range)	34.8 (1.6–99.2)	14.9 (0.4–297.4)	0.049	0.026	1.005 (1.001–1.009)
Platelets, ×10^9^/l (median; range)	57 (11–257)	47 (8–351)	0.549	0.917	1.000 (0.996–1.004)
Bone marrow blast percentage (median; range)	63 (30–98)	73 (30–100)	0.659	0.528	1.003 (0.993–1.013)
Transplant (%)	21.1	44.8	<0.001	0.089	0.671 (0.423–1.063)
Poor molecular risk (%)	36.8	24.0	0.002	<0.001	1.620 (1.266–2.072)

### Methylation analysis of CD37 gene in AML

**Figure 4:**
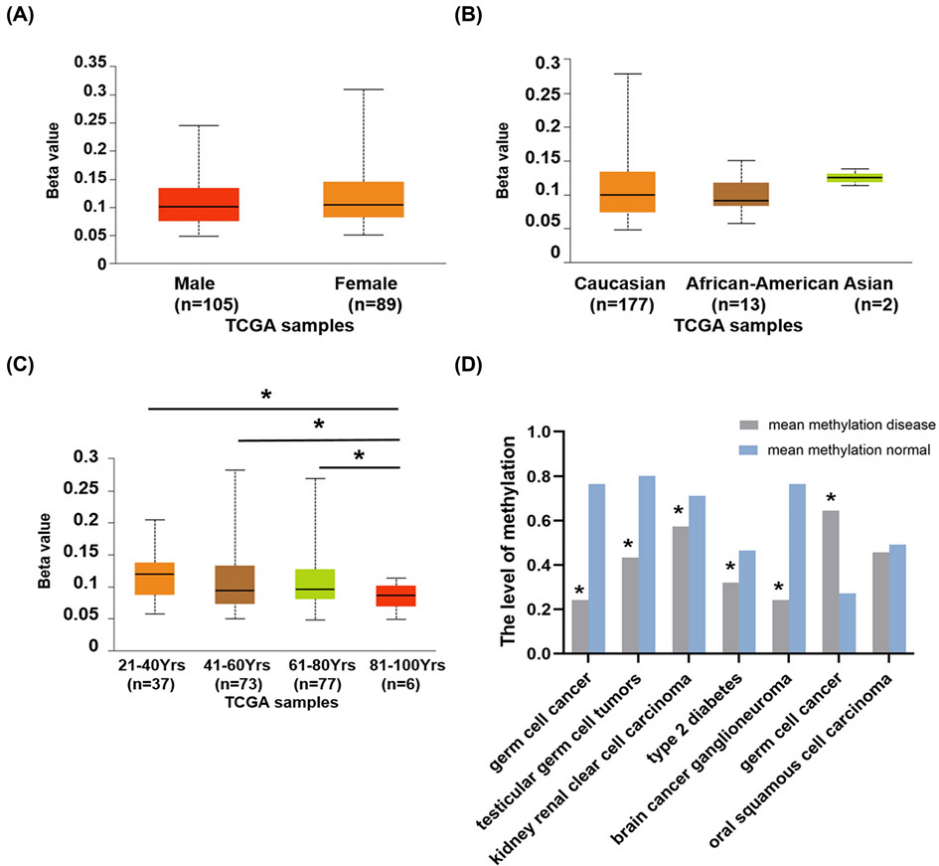
The methylation level of CD37 in AML CD37 promoter methylation profile based on (**A**) gender, (**B**) race, (**C**) age. (**D**) The level of CD37 methylation in different cancers.

In order to find the main causes of aberrant expression of CD37 in AML, we used DiseaseMeth database as well as UALCAN. We found that CD37 gene is hypermethlated in germ cell cancer, but hypomethlated in testicular germ cell tumors, kidney renal clear cell carcinoma, type 2 diabetes and brain cancer ganglioneuroma (Table 4). In AML, patients aged 81–100 have significantly lower levels of CD37 promoter methylation compared with patients aged 21–40, 41–60 and 61–80 (*P*=7.80E-04, *P*=3.02E-03, *P*=1.18E-03). No significant differences in gender and race (Figure 4).

**Table 4:** Methylation of CD37 in various cancers

Disease	Mean methyldisease	Mean methylnormal	*P*-value	Methyltype
Germ cell cancer	0.645	0.273	6.958e-04	Hypermethylation
Kidney renal clear cellcarcinoma	0.575	0.713	0.000e+00	Hypomethylation
Trisomy 18	0.479	0.417	1.396e-04	-
Oral squamous cell carcinoma	0.458	0.492	1.560e-01	-
Testicular germ tumors	0.434	0.802	0.000e+00	Hypomethylation
Type 2 diabetes	0.321	0.465	2.218e-10	Hypomethylation
Brain cancer ganglioneuroma	0.242	0.766	4.115e-05	Hypomethylation

### CD37 co-expression genes in AML

**Figure 5:**
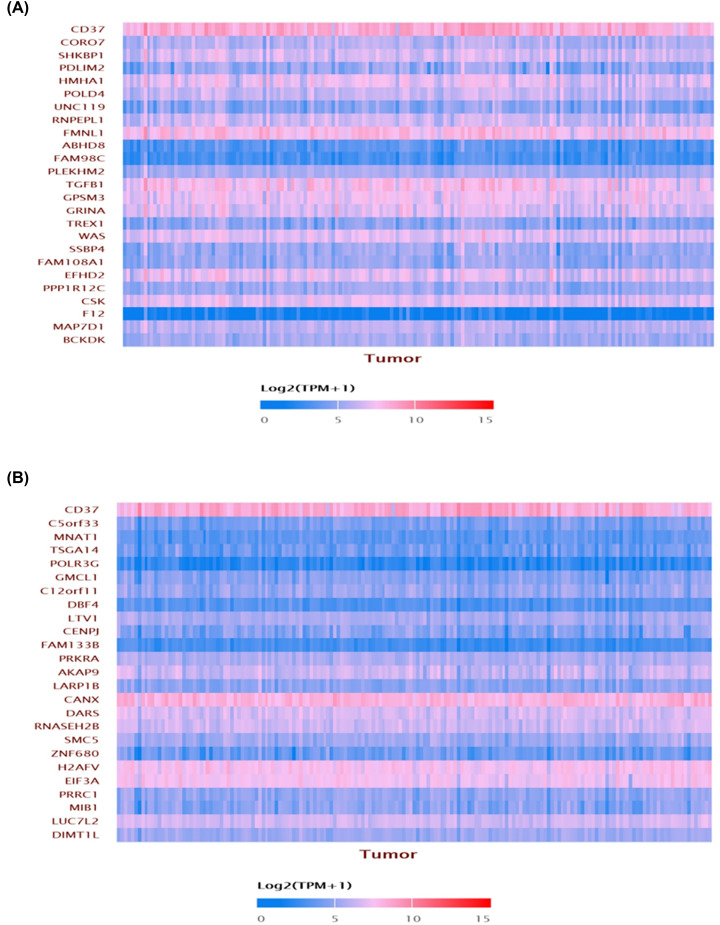
The CD37 co-expression genes by UALCAN (**A**) Genes positively correlated with CD37 expression in AML. (**B**) Genes negatively correlated with CD37 in AML.

The genes co-expressed with CD37 were obtained in AML from the UALCAN database. CD37 is positively co-expressed with CORO7, SHKBPI, PDLIM2, HMHA1, POLD4, UNC119, RNPEPL1, FMNL1, ABHD8, FAM98C etc. (Figure 5A), and negatively with C5orf33, MNAT1, TSGA14, POLR3G, GMCL1, C12orf11, DBF4, LTV1, CENPJ, FAM133B etc. (Figure 5B).

### CD37 function prediction and pathway analysis in AML

**Figure 6:**
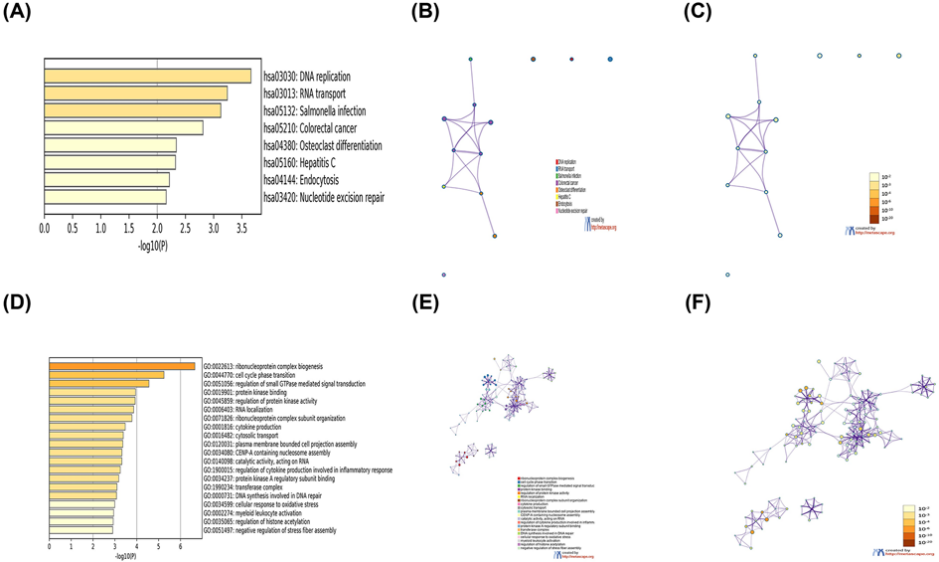
The functions of CD37 and genes significantly associated with CD37 gene alterations (**A**) Heatmap of KEGG enriched terms colored by *P*-values. (**B**) Network of KEGG enriched terms colored by cluster ID. (**C**) Network of KEGG enriched terms colored by *P*-value. (**D**) Heatmap of GO enriched terms colored by *P*-values. (**E**) Network of KEGG enriched terms colored by cluster ID. (**F**) Network of GO enriched terms colored by *P*-value, where terms containing more genes tend to have a more significant *P*-value.

To further understand the role of CD37 in AML, we made a list of genes which included 200 genes that were the most correlated with CD37 according to Pearson correlation coefficient in UALCAN. Subsequently, we analyzed the list using the Gene Ontology (GO) and Kyoto Encyclopedia of Genes and Genomes (KEGG) tools in Metascape (Figure 6). The top 20 GO enrichment items were classified into biological process group, molecular function group and cellular component group. The biological process that these genes were involved in were ribonucleoprotein complex biogenesis, cell cycle phase transition, regulation of small GTPase-mediated signal transduction and so on. The molecular function for these genes were enriched in protein kinase binding, catalytic activity, protein kinase A regulatory subunit binding. The cellular component was mainly associated with terms related to transferase complex. The top eight KEGG pathways for these genes are DNA replication, RNA transport, Salmonella infection, colorectal cancer, osteoclast differentiation, hepatitis C, endocytosis and nucleotide excision repair.

### Protein–protein interaction network analysis

**Figure 7:**
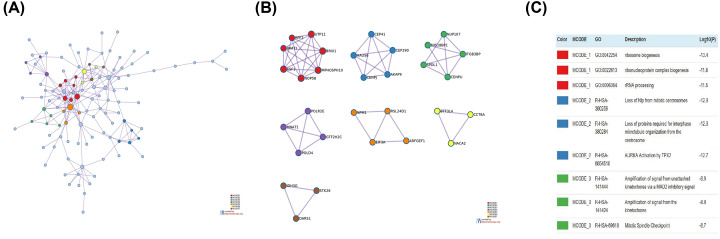
PPI network and MCODE components identified related to genes significantly associated with CD37 gene alterations (**A**) PPI network of proteins. (**B**) Modules selected from PPI network using MCODE. (**C**) The description of each MCODE component.

The protein–protein interaction (PPI) topology analysis was performed in order to systemically understand the functions of CD37 gene. The PPI network and Molecular Complex Detection (MCODE) components identified in the gene lists are shown in Figure 7. The seven most significant MCODE components were extracted from the PPI network. After pathway and process enrichment analysis was independently applied to each MCODE component, the results showed that biological function was mainly related to ribosome biogenesis, loss of NIp from mitotic centrosomes, loss of proteins required for interphase microtubule organization from the centrosome, AURKA activation by TPX2, ribonucleoprotein complex biogenesis, rRNA processing.

### Survival analysis and expression profile of key genes

**Figure 8:**
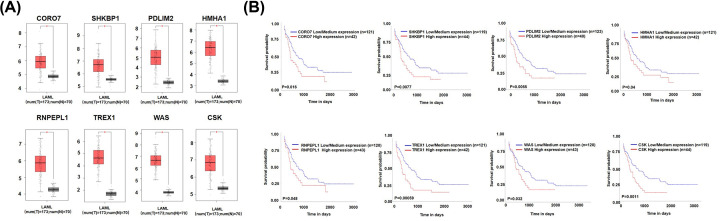
The expression level and survival analysis of key genes (**A**) The expression profile of key genes. (**B**) Survival analysis of key genes. *P<0.05 versus control.

Based on these correlation genes, eight genes were significantly associated with disease prognosis including CORO7, SHKBP1, PDLM2, HMHA1, RNPEPL1, TREX1, WAS, CSK (Figure 8). Furthermore, they are all highly expressed in AML patients, and high expression is associated with poor prognosis.

## Discussion

CD37 belongs to the members of tetraspanin superfamily, which have reached nearly 20 genes since its discovery in 1990 [4]. CD37 is absent or weakly expressed on normal T cells, monocytes and neutrophils, and is not detected on natural killer (NK) cells, platelets and erythrocytes. Thus, CD37 is considered to be a lineage-specific marker of human B cells and stands for a worthy therapeutic target in NHL. Additionally, the preclinical and early clinical studies suggested that anti-CD37 agents were useful in the treatment of CLL [21,22]. In this study, we found that CD37 is highly expressed in AML and CD37 high expression is associated with poor outcome in the patients.

We observed the expression profiling of CD37 in different types of cancers, and CD37 expression is higher in breast invasive carcinoma, cervical squamous cell carcinoma and endocervical adenocarcinoma, cholangiocarcinoma and DLBCL, but lower in adrenocortical carcinoma, bladder urothelial carcinoma, colon adenocarcinoma compared with normal adjacent tissues (Figure 1A). CD37 is significantly higher in AML. Analysis of the TCGA data showed that high CD37 expression is associated with shorter OS and DFS in AML, even if M3 is removed. These results indicated that high expression of CD37 might be a predictor of poor prognosis.

In line with these observations, Pereira et al. reported that CD37 was overexpressed in AML, and anti-CD37 IgG2 antibody exhibited potent antitumor efficacy in preclinical models of AML as well as patient-derived models [23]. However, another finding published by de Winde et al. implicated that loss of CD37 drives tumor progression via constitutive activation of the IL-6 signaling pathway [8]. In previous study, Debio 1562, a potent antibody–drug conjugate directed at CD37, was demonstrated to suppress the proliferative capacities of AML cell lines, including OCI-AML3, THP-1 and primary AML samples. Moreover, the mice engrafted with the THP-1 cell line showed a survival advantage and elimination of the disease on the basis of histopathology. Although the mechanism is unclear so far, our findings may help to provide some clues. According to our KEGG and GO enrichment results, we speculated that high CD37 blocks cell cycle progression by affecting DNA replication, thereby inhibiting AML cell proliferation.

In recent decades, mAbs have emerged as a potent and effective therapy for human malignancies [24]. For instance, the chimeric anti-CD20 antibody, IDEC-C2B8, also known as rituximab, has favorable clinical responses and became the first mAb approved by the FDA for use in human cancers [25,26]. In AML, CD33, CD123, CD44 and so on are potential targets [27–29]. Pagel et al. have reported a phase I study of an anti-CD37 smip protein in relapsed and/or refractory NHL patients [30]. Stilgenbauer et al. has reported human experience with anti-CD37 antibody in CLL, and indicated that it was a valid treatment with acceptable tolerability and notable efficacy, especially in difficult-to-treat patients with poor-risk features including del(17p) or TP53 mutations [31]. More importantly, Pereira et al. found that CD37 was also well expressed in AML, and an antibody–drug conjugate that targeted CD37-induced cytotoxicity, apoptosis and cell cycle arrest in AML cell lines [23]. This property makes the CD37 molecule an attractive target for immunotherapy. Besides, further analyses of its clinical effects and its combination with other therapeutics would bring about new insights into the mechanism of its action and strategies to improve its effects.

Additionally, our study has its limitations. First, all patients came from one single institution, which means further validations from multicenter and large sample size cohorts are needed. Second, the lack of original data to confirm our findings. Third, the mechanism about CD37 high expression in AML was associated with poor prognosis is unclear, but we believe it deserves further study in the future.

In summary, improving outcomes for AML patients, in particular those with refractory/relapsed AML, remains an ongoing challenge. As evidenced by the clinical success of rituximab, antibody-based therapies are of great importance in the management of a number of hematological malignancies. Our data suggested the oncogenic effect of CD37 high expression in AML and targeting CD37 might be a potential approach for AML therapy, which is valuable to be further explored in future.

## Conclusion

Our current study revealed the important role of CD37 gene in clinical outcomes in AML patients, and provided the rationale to identify CD37 as a valuable biomarker and develop strategies to target CD37 for AML therapy.

## Data Availability

All data generated or analyzed during the present study are included in this published article.

## Abbreviations

AML: acute myeloid leukemia
ATRA: all-*trans* retinoic acid
AURKA: aurora kinase A
CCLE: The Cancer Cell Line Encyclopedia
CLL: chronic lymphocytic leukemia
DFS: disease-free survival
DLBCL: diffuse large B-cell lymphoma
FDA: Food and Drug Administration
GEPIA: Gene Expression Profiling Interactive Analysis
GO: Gene Ontology
KEGG: Kyoto Encyclopedia of Genes and Genomes
MCODE: Molecular Complex Detection
NHL: non-Hodgkin's lymphoma
OS: overall survival
PPI: protein–protein interaction
RARα: retinoic acid receptor α
TCGA: The Cancer Genome Atlas
TPM: transcript per million
